# Omega-3 Polyunsaturated Fatty Acids: The Way Forward in Times of Mixed Evidence

**DOI:** 10.1155/2015/143109

**Published:** 2015-08-02

**Authors:** Karsten H. Weylandt, Simona Serini, Yong Q. Chen, Hui-Min Su, Kyu Lim, Achille Cittadini, Gabriella Calviello

**Affiliations:** ^1^Division of Medicine, Department of Hepatology, Gastroenterology and Metabolism, Rudolf-Virchow-Hospital, Charité University Medicine, 13353 Berlin, Germany; ^2^Lipid Clinic, Experimental and Clinical Research Center (ECRC), Max-Delbrück-Center for Molecular Medicine and Charité University Medicine, 13353 Berlin, Germany; ^3^Institute of General Pathology, Catholic University School of Medicine, 00168 Rome, Italy; ^4^The Synergistic Innovation Center for Food Safety and Nutrition, State Key Laboratory of Food Science and Technology, and School of Food Science and Technology, Jiangnan University, Wuxi, Jiangsu 214122, China; ^5^Department of Cancer Biology, Wake Forest School of Medicine, Winston-Salem, NC 27157, USA; ^6^Department of Physiology, National Taiwan University College of Medicine, Taipei 100, Taiwan; ^7^Department of Biochemistry, School of Medicine, Cancer Research Institute, Infection Signaling Network Research Center, Chungnam National University, Daejeon 301-747, Republic of Korea; ^8^Research Center for Biotechnology Applied to Cosmetology, Catholic University School of Medicine, 00168 Rome, Italy

## Abstract

Almost forty years ago, it was first hypothesized that an increased dietary intake of omega-3 polyunsaturated fatty acids (PUFA) from fish fat could exert protective effects against several pathologies. Decades of intense preclinical investigation have supported this hypothesis in a variety of model systems. Several clinical cardiovascular studies demonstrated the beneficial health effects of omega-3 PUFA, leading medical institutions worldwide to publish recommendations for their increased intake. However, particularly in recent years, contradictory results have been obtained in human studies focusing on cardiovascular disease and the clinical evidence in other diseases, particularly chronic inflammatory and neoplastic diseases, was never established to a degree that led to clear approval of treatment with omega-3 PUFA. Recent data not in line with the previous findings have sparked a debate on the health efficacy of omega-3 PUFA and the usefulness of increasing their intake for the prevention of a number of pathologies. In this review, we aim to examine the controversies on the possible use of these fatty acids as preventive/curative tools against the development of cardiovascular, metabolic, and inflammatory diseases, as well as several kinds of cancer.

## 1. Introduction

Over the past three to four decades, a considerable body of literature has been published indicating possible health benefits of an increased dietary intake of long-chain omega-3 polyunsaturated fatty acids (LC-omega-3 PUFA). Their beneficial effects have been reported for a number of disorders, including cardiovascular [1, 2], neurodegenerative [3, 4], neuropsychiatric [5, 6], and inflammatory diseases [7], as well as for some cancer types (mainly colorectal, mammary, and prostatic cancer) [8–10]. During this time period, a large number of preclinical* in vitro* and* in vivo* studies have been performed which, rather unanimously, demonstrated the potential of omega-3 PUFA as preventive and therapeutic agents against hypertriglyceridemia, cardiac arrhythmia, inflammation, and proliferation, and many experimental studies have succeeded in elucidating and clarifying many of the biological and molecular mechanisms underlying these effects [11–17]. Also, for application in humans, consensus was reached on the efficacy of these fatty acids (FA) in the secondary prevention of some cardiovascular diseases, since the results of a large and well performed clinical study [18] had clearly and strongly demonstrated beneficial effects linked to their intake. As a consequence, omega-3 PUFA have been approved as preventive and therapeutic tools in the management of several cardiovascular disorders, as well as for treatment of severe hypertriglyceridemia by a number of health agencies throughout the world, and a number of guidelines were published recommending omega-3 PUFA supplementation (see Section 3). Cardiologists and other physicians began to prescribe them routinely [19]. Moreover, as diet has always been an important topic for the popular press, recommendations have reached large segments of population, so that over-the-counter omega-3 PUFA supplements have become the most sold supplements worldwide [20].

In contrast to this remarkable story of research performance and dissemination, many human studies investigating the effect of an increased intake of omega-3 PUFA against noncardiovascular diseases were often not in keeping with data obtained in preclinical studies, and therefore consensus recommendations have not yet been made regarding the usefulness of omega-3 PUFA for the cure or prevention of inflammatory, neoplastic or neurologic disorders.

Some reports question the effectiveness of omega-3 PUFA in cardiovascular prevention [21–24], and others argue that an increased intake of these compounds could induce or exacerbate some neoplastic pathologies [25–27]. Where does this leave the field of omega-3 PUFA research and application? We will try to address this pertinent question exploring in Section 2 our body's need for omega-3 PUFA to satisfy physiological needs and to prevent the development of several kinds of diseases, in Section 3 the history and present status of omega-3 PUFA in cardiovascular diseases, in Section 4 inflammatory diseases, and in Section 5 colorectal and prostate cancer. We will describe in each of these sections the controversies arisen, and in Section 6 we will consider and critically analyze all the possible reasons for these discrepancies.

## 2. Omega-3 PUFA Insufficiency/Deficiency and Current Recommended Intakes

Alpha-linolenic acid (ALA), an 18-carbon omega-3 essential FA, is the precursor of eicosapentaenoic acid (EPA) and docosahexaenoic acid (DHA). The term “essential” indicates that ALA cannot be synthesized by humans and therefore must be entirely acquired from exogenous sources. Evidence for the essentiality of ALA was first provided by a study showing that ALA supplementation reversed the abnormal neurologic signs observed in a 6-year-old girl who suffered from sensory loss and visual complications [28]. Following consumption, most of the ALA is catabolized via *β*-oxidation for energy generation, and a small proportion of it undergoes conversion to produce another two potent members of omega-3 PUFA family: EPA and DHA [29]. The conversion rates of ALA to EPA and DHA in humans are estimated at 8–20% and 0.5–9%, respectively [30]. Owing to the fact that EPA and DHA can be synthesized in the body from ALA, these two FA do not meet the definition of essential FA* per se*. However, as this conversion is not efficient enough to satisfy health requirements, EPA and DHA are also considered essential FA (or conditionally essential FA). In addition, although not conclusive, the benefits associated with ALA seem to stem mainly from EPA and DHA, and as major consequence of ALA deficiency it appears that EPA and DHA are not adequately produced [31, 32]. The clinical features of omega-3 PUFA insufficiency include impaired growth, skin lesions, infertility, kidney abnormalities, fatty liver, polydipsia, increased susceptibility to infections, reduced learning, and impaired vision [33, 34]. Yet, these symptoms are nonspecific and may also result from the dysregulation in omega-6 PUFA homeostasis [35]. For example, the skin lesions present in patients with atopic eczema have been associated with poor omega-6 PUFA intake [36].

An increased intake of omega-3 PUFA, especially the long-chain omega-3 PUFA EPA and DHA, could reduce the tissue omega-6/omega-3 ratio to a level that probably existed during millions of years of human evolution [37]. This ratio dramatically increased in recent millennia due to deep changes in dietary habits following the transition from the hunter-gatherer lifestyle to agricultural societies. This change could therefore be one of the crucial factors leading to the rise of the so-called diseases of civilization, further skewed towards omega-6 PUFA by the agricultural revolution in the 19th century and the massive use of corn (with its high omega-6 PUFA content) in western societies during the 20th century.

Dietary recommendations for omega-3 PUFA, previously focused on ensuring their sufficient intake to prevent deficiency, are increasingly seeking to define an “optimal” intake to reduce the risk of developing chronic diseases, in particular cardiovascular diseases (CVD). To date, there is no uniform scientific guideline on the ideal omega-3 PUFA intake. Nutritional guidelines have been published by several governments (France, Belgium, Netherlands, New Zealand, and Australia) and health organizations (Food and Agriculture Organization (FAO), American Dietetic Association). The most popular recommendations on omega-3 PUFA intake are those published by the American Heart Association (AHA), the UK Committee on Medical Aspects of Food Policy, the World Health Organization (WHO), and the European Food Safety Authority (EFSA). Recommendations for omega-3 PUFA (ALA, EPA, and DHA, individually or combined) for adults according to various recently published dietary guidelines are shown in Table 1, whereas Table 2 shows recommendations for infants and children [38–53]. Dietary recommendations for EPA + DHA range from 250 to 1000 mg/day for adults and from 40 to 250 mg/day for infants older than six months and for children and adolescents.

## 3. Omega-3 PUFA Efficacy in the Cardiovascular Field

The predominant field of omega-3 PUFA research in the past decades has been that of cardiovascular medicine and prevention. This is the field in which the hypothesis of a protective effect of omega-3 PUFA was founded [69], and a large clinical study establishing a benefit was the basis for approval of omega-3 PUFA as secondary prevention in patients after myocardial infarction [18]. The GISSI-Prevenzione trial was performed as an open-label trial in the 1990s in 11324 patients shortly after myocardial infarction. Participants in the omega-3 PUFA group received 1 g/d omega-3 PUFA (ratio of EPA/DHA 1 : 2, with a total amount of EPA + DHA of approximately 850 mg) for 3.5 years. There were no systematic fatty acid measurements performed in the participants, but, as an indication of omega-3 PUFA uptake, triglycerides were lower in the omega-3 PUFA group. Treatment with omega-3 PUFA significantly lowered the risk of death, including cardiovascular death [18]. This area has seen some good examples of translational research, particularly with regard to the antiarrhythmic effects attributed to omega-3 PUFA. Leaf [70, 71] was a pioneer in the field of omega-3 PUFA research showing that these FA can block proarrhythmogenic effects of adrenal agents, calcium, and other substances in isolated cardiomyocytes, continuing with studies in animals [72], and finally concluding his research in a large human multicenter trial [54]. The concept of cardioprotection through omega-3 PUFA supplementation has been firmly engrained into the canon of medical knowledge and clinical thinking, as is evidenced by the AHA guidelines recommending intake of omega-3 PUFA [73].

However, while preclinical data regarding the antiarrhythmic effect of omega-3 PUFA were strongly beneficial, the double-blind intervention study by Leaf et al. [54] does not show a clear benefit. A relevant problem of the study was the high noncompliance rate (35% of enrollees, similar in fish oil and olive oil groups), probably due to the high capsule load (2600 mg fish oil per day). Treatment with daily fish oil or olive oil for 12 months in patients with implanted cardioverter/defibrillators (ICDs) did not lead to a significant difference for the primary end point (time to first ICD event for ventricular tachycardia (VT) or death from any cause). However, there was a significant effect in participants who were at risk of fatal ventricular arrhythmias and, after staying on protocol for at least 11 months, showed a significant risk reduction of 38%. Phospholipid fatty acid content of red blood cells was compared in a subset of participants in the study, and omega-3 PUFA content increased significantly from 3.4% to 7.6% of total fatty acids, while it remained at 3.5% in the olive oil control group.

There are two more randomized, double-blind, placebo-controlled trials with omega-3 PUFA supplementation performed in patients with ICDs and at high risk of high-grade arrhythmic events. In one, 200 patients with an ICD and a recent episode of sustained VT or ventricular fibrillation (VF) received either 1300 mg/d omega-3 PUFA or placebo and were followed up for 2 years. While patients on fish oil had an increase in the mean percentage of omega-3 PUFA in red blood cell membranes from 4.7% at baseline to a steady-state maximum of 8.3% at 3 months, there was no protection from VT/VF events [59]. The third study did not find a significant advantage for omega-3 PUFA supplementation in ICD patients receiving 2 g/d of fish oil (*n* = 273) versus placebo (*n* = 273) for one year with the primary outcome of an ICD event or death occurring in 30% patients taking omega-3 PUFA versus 33% of those on placebo [60].

In addition to these high-quality data sets showing uncertainty regarding the antiarrhythmic effect of omega-3 PUFA, several large studies followed up on the initial positive GISSI-P results. The GISSI-HF investigated the effect of 1 g/d omega-3 PUFA in patients with heart failure in a randomised, double-blind, placebo-controlled trial with 6975 participants. Over approximately 4 years there was a small significant advantage in the omega-3 PUFA group for death from any cause and admission to hospital for cardiovascular reasons with a number needed to treat of 56 to prevent one death [55].

A multicenter, double-blind, placebo-controlled trial with 4837 patients after a myocardial infarction tested 40 months of fatty acid supplementation in the form of margarines, either adding a small amount of approximately 400 mg/d EPA + DHA, 2 g/d of ALA, both EPA + DHA and ALA, or placebo fat. Neither EPA-DHA nor ALA reduced the primary end point of a major cardiovascular event. Fatty acids were measured in plasma cholesteryl esters of subgroups, and supplementation with EPA + DHA increased EPA by 53.3% and DHA by 28.6%, as compared with placebo after 20 months. Unfortunately, fatty acid composition of red blood cell membranes was not measured in this study [21].

Another randomized, placebo-controlled, double-blind, multicenter trial in 3851 patients with 1 g/d omega-3 PUFA supplementation for one year in patients after acute myocardial infarction also demonstrated no benefit of omega-3 PUFA [58].

A third large study with 2501 participants found that administration of 600 mg/d omega-3 PUFA increased plasma concentrations of EPA + DHA by 37% compared with placebo but also had no significant effect on major vascular events in patients with ischaemic heart disease or ischaemic stroke [56].

Based on these and several smaller intervention studies assessing mostly relatively low-dose omega-3 PUFA supplementation, two thorough metastudies concluded that there is no convincing evidence for a preventive effect of omega-3 PUFA in patients with cardiovascular disease [22, 62].

The ORIGIN study [23] is the most recent large study that failed to show a clear benefit of omega-3 PUFA supplementation for cardiovascular health. This study, conducted in a patient population with dysglycemia, led many physicians to conclude that the relationship between dietary omega-3 and cardiovascular health needs to be viewed with caution. In this double-blind study 12536 dysglycemic patients at high risk for cardiovascular events were assigned to either 900 mg/d omega-3 PUFA or placebo. The primary outcome was death from cardiovascular causes during a median follow-up of 6.2 years, and there were no significant differences between the two treatment groups for this primary parameter as well as for a range of secondary parameters. It is noteworthy that the significant decrease of triglycerides is an indicator of effective omega-3 PUFA exposure in the verum group. However, unfortunately, fatty acid levels in the blood, particularly in the red blood cell fraction, were not evaluated.

Nutritional research, especially the clinical trials investigating different dietary regimes, has always been surrounded by controversy. The role of dietary fat in obesity and associated clinical conditions is a prime example [74], with ongoing uncertainty about the role of a low-fat diet in the battle against cardiovascular disease [61]. Recent recommendations by the AHA and the medical bodies in the US acknowledged also a protective effect of the omega-6 PUFA [53], and more studies have added complexity and controversy to the role of different FA classes (saturated fats, monounsaturated olive oil, polyunsaturated omega-6, and omega-3 PUFA), challenging not only the hypothesis of beneficial effects associated with unsaturated fatty acids but also the paradigm of the damaging effects of saturated fatty acids [61, 75].

What can we learn from all this regarding the potential cardiovascular benefits of omega-3 PUFA? It is difficult from today's perspective to adequately assess the conditions of the 70s observations regarding the omega-3 effects in the Greenland population, and the arguments of inadequate data validity in these studies made by Fodor et al. [24] are plausible giving the remoteness of many Greenland communities and problems with medical service and adequate documentation in these areas.

While the protective cardiovascular effect of omega-3 PUFA was not detected in several large recent high-quality studies, there are particularly two high-quality intervention studies, both of them open-label studies though, showing a clear benefit of omega-3 PUFA, the GISSI-P study [18] and the JELIS study demonstrating a protective effect of an EPA preparation combined with a statin medication [57]. This large open-label study performed on 18645 Japanese patients assigned them to randomly receive either 1800 mg/d of EPA daily plus statin or statin only. The primary endpoint of a major coronary event was significantly reduced in the EPA group with a 19% relative reduction. However, sudden cardiac death and coronary death did not differ between the groups. Plasma EPA was measured in this study and in patients still on supplement after 5 years of observation plasma EPA concentration was increased by 70% in the EPA group.

In conclusion, many high-quality preclinical (observational as well as interventional) and clinical studies have been performed to assess the potential cardiovascular benefit of omega-3 PUFA. A recent large metastudy has again analyzed and summarized these studies and found no clear benefit of omega-3 PUFA to protect from coronary artery disease in observational studies as well as in randomized, controlled trials [61]. This metastudy also explored the role of other fatty acids and concluded that the current evidence does not clearly support cardiovascular guidelines encouraging either high consumption of omega-3 or omega-6 PUFA, or even low consumption of saturated fats.

Cardioprotective effects of dietary omega-3 PUFA might be confounded by the use of cholesterol-lowering medications: in the GISSI-P study only 5% of participants were on a cholesterol-lowering drug [18], which is in strong contrast to the ORIGIN omega-3 PUFA study, where approximately half the patients were on a statin [23]. Statins have been hypothesized to interfere with the protective effect of omega-3 PUFA [76].

Modifications in the observed omega-3 effects might also be due to changes in the diet over the last 30–40 years, in which the hypothesis of omega-3 PUFA as beneficial dietary components has been propagated, and therefore background levels of omega-3 PUFA in the participants of more recent studies might be higher than several decades ago. This should be addressable by (comparable) measurements of omega-3 PUFA that, unfortunately, have been performed only sporadically in the studies carried out to date. Baseline omega-3 PUFA levels are lacking from most of the discussed large studies investigating omega-3 PUFA interventions. Several studies have suggested that erythrocyte EPA and DHA content (measured by a certified method as “omega-3 index”) may better reflect the omega-3 PUFA status in patients than measurement in plasma and white blood cells [77, 78], and the low omega-3 index was described to be associated with higher mortality in patients with cardiovascular disease. However, so far, there has been no single well-accepted and uniformly performed blood test for the omega-3 PUFA status in human studies, complicating the comparison and interpretation of different studies. The current state of evidence for the effects of omega-3 PUFA in human studies regarding cardiovascular diseases and the contrasting outcomes have been summarized in Table 3. Additional possible reasons for the controversies in the field are reported in Section 6.

Last but not least, it should be noted that the effect of omega-3 PUFA in the cardiovascular field can also be viewed in the context of their role as precursors of highly potent physiologically beneficial lipid mediators [79, 80]. A cytochrome P450 epoxygenase-dependent metabolite of EPA, namely, 17(*R*),18(*S*)-epoxyeicosatetraenoic acid (17(*R*),18(*S*)-EETeTr), was found to exert negative chronotropic effects and can protect neonatal rat cardiomyocytes against Ca2+-overload [81]. Another EPA-metabolite, 18-hydroxyeicosapentaenoic acid (18-HEPE), was recently shown to inhibit macrophage-mediated proinflammatory activation of cardiac fibroblasts* in vitro*, while* in vivo* administration of 18-HEPE led to resistance to pressure overload-induced maladaptive cardiac remodeling in mice [82].

## 4. Omega-3 Efficacy in Inflammatory Diseases

Research data accumulated to date indicate that omega-6 PUFA play a significant role in the biology of inflammation. Work on the biochemistry of omega-6 led to the identification of prostaglandins and leukotrienes as key players in the physiology of inflammation [118]. Moreover, this work elucidated the cascade of the molecular mediator system arising from the omega-6 PUFA arachidonic acid and led to mechanistic understanding and further development of the most widely prescribed drug class worldwide, the cyclooxygenase- (COX-) inhibitors [119].

Early work on omega-3 PUFA identified the antagonism between omega-6 and omega-3 PUFA. It was postulated that, by competitive inhibition of COX and other enzymes, omega-3 PUFA could serve as anti-inflammatory agents. Indeed, it has been found that a high omega-3 intake was associated with a lower risk of inflammatory disease mortality [120].

Many animal studies demonstrated the anti-inflammatory effect of omega-3 PUFA. As far as animal models of colitis are considered, EPA-ethyl or DHA-ethyl ester supplementations (1% w/w in the diet, given for 10 days prior to treatment) were reported to reduce colitis obtained in dextran sodium sulfate- (DSS-) treated mice [121], as demonstrated by their ability to inhibit the expression of several proinflammatory cytokines. Similar results were obtained also by supplementing a FO enriched diet (concentration not specified, dietary n:6/n:3 ratio: 1.5, for 7–11 weeks) in a severe combined immunodeficient mouse model of colitis [122]. In agreement with that, a FO enriched diet (containing 1% FO providing 0.5 g EPA + DHA g/day, for 5 weeks prior to infection) was shown to attenuate infection-induced colitis in mouse infected with* Citrobacter* [123]. Furthermore, also a parenteral lipid emulsion enriched in omega-3 PUFA (10% FO, 0.5 mL/h for 7 days) was found to reduce acetic acid-induced colitis in rats [124].

However, opposite results have also been obtained and extensively and recently reviewed by Fenton et al. [125]. In particular, dietary FO (8% w/w in the diet, for 5 weeks, starting one week prior to treatment) was shown to increase DSS-induced colitis [126]. Moreover, diets containing 2.25–6.00% FO (containing 59% DHA + EPA, for 8 weeks after infection) were recently found to induce severe colitis and epithelial dysplasia in mice infected with* Helicobacter hepaticus* [127]. Furthermore, FO (7% FO w/w in the diet, for 12 weeks) was found to exacerbate colitis and colitis-associated premalignant lesions [128] in IL-10 −/− transgenic mice, spontaneously developing enterocolitis [129]. In contrast, Chapkin et al. [130] showed that a FO enriched diet (4% FO w/w in the diet, for 10 weeks) fed to the same IL-10 −/− transgenic mice reduced the clinical score of both spontaneous and NSAID-induced colitis.

Interestingly, however, when at least the uncertainty related to variable intakes of FO was overcome by using the* Fat-1* mouse model of endogenously increased omega-3 PUFA [131], the inflammation-dampening effect of an increased omega-3 PUFA tissue content was consistently observed in the murine models of DSS-induced colitis [132, 133]. Moreover, when* Fat-1* mice were also treated with the carcinogen azoxymethane prior to DSS, an enhanced ability to resolve colitis and a reduced number of colonic adenocarcinomas per mouse were observed [134, 135]. In the* Fat-1* mouse model a reduced D-galactosamine/lipopolysaccharide- (D-GalN/LPS-) induced hepatitis [136] and cerulein-induced pancreatitis [137] were also observed.

As concluded by Fenton et al., it is particularly in the context of studies with infectious agents that deleterious effects of omega-3 PUFA were observed [125], and these authors argue that the same (auto)inflammation-dampening and cardioprotective properties of the omega-3 PUFA might be responsible for an ineffective immune response against pathogens and even decreased immune surveillance against tumor cells. However other data do not support this view of a uniformly immunosuppressant effect of omega-3 PUFA: a recent study shows that omega-3 PUFA can actually increase B-cell function and thus humoral immunity [138].

Also the clinical data from intervention trials with omega-3 appear to be controversial. Initial results in Crohn's disease and ulcerative colitis [63, 66] found a benefit. However, later studies showed a negligible impact of omega-3 PUFA on the relapse rate in Crohn's disease [65] as well as in ulcerative colitis; the studies performed in inflammatory bowel disease are reviewed in a recent metastudy, concluding that total data quality so far is insufficient for clear recommendations [68]. Data from acute pancreatitis in humans indicated some benefits [67]; however, there are no data from chronic pancreatitis, and the recently published Welcome Trial data regarding the use of omega-3 PUFA in nonalcoholic steatohepatitis (NASH) do not show an overwhelming benefit of supplementation in the intention-to-treat (ITT) analysis, even though there was a statistically significant linear correlation between decreased liver fat content and increased DHA enrichment [64]. The current state of evidence for the effects of omega-3 PUFA in human studies regarding inflammatory diseases and the contrasting outcomes have been summarized in Table 4. Possible reasons for the controversies in the field are reported in Section 6.

Experimental data from recent years are currently reshaping omega-3 PUFA research: since the initial description of resolvins [139] and the characterization of resolvin E1 [140], a wealth of data on this new class of omega-3 PUFA-derived lipid mediators has been published (reviewed in [80]). It is now clear that omega-3 PUFA not only play a role as competitive antagonists of the COX and lipoxygenase (LOX) enzymes, but also are substrates for potent lipid mediators themselves. It has been shown that a high abundance of these FA as substrates can significantly change the lipidome and eicosanome, given that they are efficiently metabolized as well [79]. For the DHA-derived D-resolvins, 17-HDHA has been characterized as a central pathway precursor/metabolite, and for the EPA-derived E-resolvins, 18-HEPE serves this role. Data from one of the authors have indicated that these might have anti-inflammatory properties themselves [141–143]. Other groups have described the effects of 17-HDHA as well [144–146], and very recently an independent role of 18-HEPE was also demonstrated in the context of a cardiac fibrosis model [82]. This is currently a very active field of research and will probably give us new tools for understanding the effects of omega-3 PUFA in the context of inflammation dampening, as well as a number of new leads for pharmacological compounds that could be used to mediate anti-inflammatory effects in the future. As these compounds can promote resolution of inflammation [147], this could lead to an entirely new approach to the treatment of chronic inflammation in the future.

Closely related to these effects regarding inflammation dampening are the experimental data regarding tumorigenesis. One of us and his group have validated a tumorigenesis inhibiting effect in the* Fat-1* mouse model with endogenously increased omega-3 PUFA for liver cancer [143] and colon cancer [135]; this fits well with data from other groups employing the* Fat-1* mouse model [134, 148, 149].

Particularly in gastrointestinal medicine, the paradigm of inflammation-associated carcinogenesis is central. It holds true for the development of esophageal carcinoma on the basis of Barrett's esophagus, stomach cancer on the basis of chronic* H. pylori* infection, liver cancer as a consequence of chronic hepatitis, cholangiocellular cancer in chronic cholangitis, pancreatic cancer as a consequence of chronic pancreatitis, and also in colorectal cancer. There are clinical data supporting the tumorigenesis inhibiting effect of omega-3 PUFA in the gastrointestinal tract, either with regard to colon cancer and the lowering of polyp formation in the colon [83, 90, 91] or with regard to hepatocellular carcinoma [150]. It is therefore conceivable that increasing omega-3 PUFA intake could be established as an easy and low-risk, high-gain intervention for the prevention of tumorigenesis. Given the very low risk of severe adverse events in comparison to interventions such as the use of the COX-inhibitor aspirin, for which similar antitumorigenesis effects are well established by now [151, 152], omega-3 PUFA supplementation becomes an attractive perspective. An ongoing study is even testing the combination of EPA and aspirin for the prevention of colon polyps [153].

## 5. Omega-3 Efficacy in Cancer

### 5.1. Mechanisms Involved in Omega-3 PUFA Anticancer Action

Most of the studies performed either* in vitro* or using animal models of cancer have demonstrated the possible preventive and therapeutic role of omega-3 PUFA against several types of cancer. These studies have also shed light on multiple molecular pathways modulated by these fatty acids in cancer cells and implicated in the regulation of several cell processes involved in cancer development and progression, such as cell proliferation, survival, differentiation, invasion, and angiogenesis. Actually, it is quite astonishing how many signaling pathways and molecular targets have been so far implicated in the antineoplastic effects of these dietary compounds [8, 15, 154–156]. However, it has been hypothesized that these fatty acids may more directly act through just a few main direct routes and consequently influence the activity/expression/levels of plenty of cellular pathways and molecules more or less specifically altered in the different cancer tissues. Actually, the topic concerning the mechanisms underlying the omega-3 PUFA antineoplastic action is very complex and beyond the focus of this review. Several exhaustive reviews have been previously written by us and others that critically analyzed the possible cell signaling pathways involved in omega-3 antineoplastic action. The readers may refer to them to have a more complete view [8, 15, 154–159].

Nevertheless, the main direct routes through which omega-3 PUFA are hypothesized to more directly act are worth mentioning here. One of these routes is related to the omega-3 PUFA increased incorporation in cell membranes, as it happens consequently to their increased dietary intake. As a result, physical-chemical alterations of molecular lipid microenvironments (rafts) take place on cell surface [160]. These modifications may in turn lead to changes in the activity/expression of membrane constituents (receptors, channels, enzymes, adapter proteins, etc.) and multiple molecular pathways placed downstream. For instance, it was recently observed [121] that the incorporation of DHA in immortalized colonocytes* in vitro* altered the lateral organization of the Epidermal Growth Factor Receptor (EGFR), thus leading to increased ligand-induced receptor dimerization and phosphorylation along with its internalization and degradation. These changes, in turn, resulted in the disruption of the EGFR-Ras-ERK1/2 signaling cascade and the inhibition of cell proliferation. Accordingly, in chronically inflamed, carcinogen-injected mice, these authors found that a DHA-enriched diet induced the same effects observed* in vitro* on the EGFR signaling pathway and also inhibited tumor development. Similarly, we recently found [161] that DHA could dislocate from colon cancer cell surface the antiapoptotic Glucose Related Protein of 78 kDa (GRP78), abnormally expressed on cancer cells plasma membranes [162], where it acts as a signaling receptor promoting proliferation and survival. Through this mechanism, DHA inhibited the ERdj5/PERK/caspase-4 and caspase-7 pathway placed downwards and induced apoptosis [161].

A second main possible direct route for the omega-3 PUFA action is related to their high peroxidability that makes them optimal substrates for oxidants inside the cells [163]. Through this route, these fatty acids may induce alterations of the cell oxidative status and modulation of oxidative stress-dependent molecular pathways related to cell proliferation, apoptosis, or inflammation.

Moreover, a third main direct route for the omega-3 PUFA action is related to their possible metabolic conversion into bioactive molecules with powerful anti-inflammatory and proresolving action (i.e., resolvins, protectins, etc.). Receptors binding specifically to both these bioactive molecules (such as ChemR23, leukotriene B4 receptor 1, LTB4R1, and G protein coupled receptor, GRP32) [164] as well as to omega-3 PUFA (such as peroxisome proliferator activated receptor *γ*, PPAR*γ*, and G protein coupled receptor, GPR120) have been recently identified [164, 165]. The signaling starting from these receptors may be translated downwards into the induction of specific cellular molecular pathways.

Finally, a very interesting possibility that may represent the field of future promising investigation in this research area is that omega-3 PUFA, through one or more of these main direct routes, may in turn affect DNA cytosine methylation, the covalent modifications of histones, or the expression of noncoding microRNA (miRNA). These effects may alter the epigenetic control of genes codifying proteins involved in cell proliferation and survival. It has been already hypothesized that alterations of epigenetic control of gene expression could be involved in the beneficial effects that omega-3 PUFA exert in immunological or neurodegenerative diseases [166–168] or in some kinds of cancer [157, 169–171].

### 5.2. Omega-3 PUFA in Colon Cancer

Dietary omega-3 PUFA have attracted considerable interest for their potential to prevent the development and progression of colorectal cancer (CRC) [8, 158, 172]. An impressive body of evidence has been obtained in preclinical studies using* in vivo* CRC models, consistently supporting the antineoplastic role of omega-3 PUFA [8, 154, 172], in spite of the high variability of the models and experimental conditions used [134, 135, 154, 173, 174]. Among all these data only very few were in conflict with a protective effect of the omega-3 PUFA [127, 175], but in these cases extremely high doses of LC-omega-3 PUFA were administered (about 3–7 g EPA + DHA/kg body weight in mouse or 12 g EPA + DHA/kg body weight in rat; for the calculation used, see [216]). These high doses may generate vast amounts of oxidized products with high prooxidant and carcinogenic activity [26].

Many* in vitro* studies [8, 154, 172] confirmed the antineoplastic activity of omega-3 PUFA and, as discussed in the previous section, allowed identification of possible biological and molecular mechanisms [154]. Remarkably, among the* in vitro* studies [176–178] are those that recently investigated the possible effects of omega-3 PUFA on colorectal cancer stem cells (CCSC). It is believed that CCSC may drive colon tumorigenesis, being principal targets of tumorigenic genetic alterations, due to their long lifespan and capacity for self-renewal. Moreover, they have also been related to cancer relapse, acquisition of chemotherapy resistance, and metastasis [179]. CCSC are characterized for lacking specific markers of colonic epithelium differentiation, such as cytokeratin 20 (CK20) or mucin 2 (MUC2), and expressing instead specific clusters of differentiation, such as CD133 or Lgr5 antigens (labeling undifferentiated cells) [180–182]. Moreover, most of cancer stem cells form spheres when cultured in serum-free conditions that are highly tumorigenic if injected in immunodeficient mice [176, 178]. Yang et al. [176] cultivated cancer stem-like cell spheres derived from SW620 colon cancer cell line and showed that both EPA and DHA (10–70 *μ*M) inhibited their growth by inducing apoptosis and that the effect was markedly increased when they acted simultaneously. Moreover, EPA and DHA enhanced the efficacy of chemotherapeutics agents such as 5-fluorouracil (5-FU) and mitomycin C. Instead, de Carlo et al. [177] found that whereas 25 *μ*M EPA inhibited the growth of the COLO 320 DM colon cancer cell line* in vitro*, it was unable to inhibit that of its CD133+ subpopulation. However, EPA upregulated CK20 and MUC2 and downregulated CD133 expression, thus indicating that it triggered the transition of CCSC to a more differentiated cancer cell phenotype. The authors hypothesized that the increased degree of CCSC differentiation induced by EPA could be strictly related to the EPA-induced sensitization of CD133+ cells to the anticancer agent 5-FU that they observed. Accordingly, Vasudevan et al. [178] found that EPA alone and especially in combination with 5-FU + oxaliplatin (FuOX) was effective in inhibiting cell growth and sphere formation, as well as in inducing apoptosis of (FuOX-) resistant HT-29 and HCT116 CRC, highly enriched in CCSC.

The confirmation in different experimental settings [176–178] that omega-3 PUFA can increase the sensitivity of colon CCSC to conventional antineoplastic drugs is very interesting, since, as reviewed by us and others previously [183–185], the most feasible potential anticancer application for these fatty acids in humans will be their possible use as adjuvants—to help increase nutrition status, as well as affort/modulate anticancer effects—in combination with conventional or single-targeted anticancer therapies.

If we turn to consider the interventional studies conducted on healthy volunteers, CRC patients, or subjects at high risk for CRC, the majority of them have concurred to demonstrate the beneficial antineoplastic effect of omega-3 PUFA (Tables 5(a) and 5(b)). The first studies were performed about twenty years ago by supplementing purified EPA + DHA or fish oil (FO). They demonstrated the efficacy of these FA in inhibiting abnormal CRC cell proliferation [83–85, 87], when given either in higher amounts (daily dose: 8-9 g EPA + DHA) for short (2 weeks) and longer periods (3–6 months) or in lower amounts (daily dose: 2.5–4 g EPA + DHA) after one month [85]. However, when the same treatment was performed in the presence of a basal high-fat diet and a low omega-3/omega-6 PUFA ratio of 0.25, no effect on rectal mucosa cell proliferation was observed [86], confirming that the efficacy of omega-3 PUFA is dependent on both the total lipid content and the omega-3/omega-6 PUFA ratio of the diet. Also Gee et al. [88] did not find any change in cell proliferation by administering 2.4 g/day EPA + DHA for 3 months, but in this case there is no available information on basal diet or dietary omega-3/omega-6 PUFA ratio. Instead, Cheng et al. [89], who treated patients at high risk for CRC with lower doses (as FO, about 500 mg EPA + DHA/day), but for a very long period (2 years), observed apoptosis induction in the mucosa adjacent to the resected CRC [89]. More recently, Courtney et al. [90] administered gastroresistant capsules containing EPA-free fatty acid (FFA, 2 g/day) and confirmed the inhibition of cell proliferation and induction of apoptosis in colonic mucosa of subjects at high risk for CRC cancer. The same treatment was recently shown [91] to reduce the number and size of rectal polyps in familial adenomatous polyposis (FAP) and to inhibit angiogenesis in patients carrying CRC liver metastases [92]. A large multicenter trial (seAFood Polyp Prevention Trial) [153] is now being conducted, supplementing the EPA-FFA formulation alone or in combination with aspirin to patients at high risk for developing colorectal adenomas. It would be interesting also to study the effect of DHA in the same formulation, since antineoplastic activities have also been reported for both fatty acids [155]. Moreover, even though EPA can be metabolically converted into DHA and DHA retroconverted into EPA, the* in vivo* efficiency of these two metabolic reactions may be substantially different [186].

The outcomes of the recent large multicentric interventional FISHGASTRO study [93, 94] showed that an extra 300 g/week fish serving for 6 months lowered significantly the serum C-Reactive Protein (CRP) concentration in all the subjects, but it did not alter the levels of several inflammation markers in fecal water and colonic biopsies or change the apoptotic rates in colonic mucosa. However, the mitotic rate decreased in the fish groups, even though the significance was not attained [93]. Still, it is possible to hypothesize that the small extra supplementation on top of an already large amount of fish consumed at baseline (corresponding to about 0.09 or 1.4 g EPA + DHA/day) was not sufficient to induce significant alterations in mucosal cell growth and survival. The reduction in serum CRP levels was confirmed in a recent trial conducted by Mocellin et al. [95] in CRC patients treated daily with FO containing 0.6 g EPA + DHA, supporting a general anti-inflammatory effect of omega-3 PUFA supplementation.

After more than twenty years of intense research, several meta-analyses and systematic reviews of the literature have been recently published on this topic. If we take into account only the most recent ones [100–102], their conclusions appear not to be in agreement. Gerber [102] updated the observations of the previous meta-analysis conducted by the FAO/OMS experts [101] to April 2011 and prudently concluded that overall, owing primarily to insufficient homogeneity of the observations, the studies analyzed provided only limited evidence suggesting a role for omega-3 PUFA in CRC prevention [102]. Wu et al. [100] considered in their meta-analysis 22 prospective cohort and 19 case-control studies performed until 2011. They more decidedly concluded that fish consumption was inversely associated with CRC. In particular, they found a significant inverse association between fish intake and rectal cancer and only a modest trend between fish consumption and colon cancer.

Since then, several other case-control and cohort studies were published. In their case-control studies, Murff et al. [96] and Habermann et al. [99] demonstrated an inverse association between an increased omega-3 PUFA dietary intake and the risk of developing colorectal neoplasms (adenocarcinomas or adenomas) [96, 99]. However, benefits were limited only to some of the subpopulations investigated. In particular, Murff et al. [96] showed that an increased omega-3 PUFA intake was associated with reduced risk of colorectal adenomas in women only, whereas Habermann et al. [99] found that an inverse association between low DHA intake and increase in CRC risk was confined to patients showing specific genetic variants that resulted in higher levels of proinflammatory mediators.

Recent cohort studies found associations only in subcohorts. Kantor et al. [97] found an inverse association between FO use and cancer risk, but only at the level of the colon (not rectum) and only in men (not in women). Moreover, the same authors [97] found that fish consumption or EPA/DHA intake was not associated with CRC risk overall but that associations were significantly modified by genetic risk, with inverse associations among low-moderate genetic risk groups and positive associations among high risk groups. The cohort study of Song et al. [98] found no association between fish/omega-3 PUFA intake and overall CRC incidence. In contrast with the Kantor et al. study [97], Song et al. [98] found an inverse association between a high LC-omega-3 PUFA consumption and rectal cancer (but not colon cancer) and even a positive association between LC-omega-3 PUFA increased intake and distal colon cancer.

The controversial findings in epidemiological studies now need clarification in the setting of large randomized clinical intervention trials, such as the one currently ongoing [153]. The current state of evidence for the effects of omega-3 PUFA in human studies regarding colorectal cancer and the contrasting outcomes have been summarized in Table 6. Possible reasons for the controversies in the field are reported in Section 6.

### 5.3. Omega-3 PUFA in Prostate Cancer

Recently, Brasky et al. [25] suggested a potential prostate cancer promoting effect of omega-3 PUFA, leading to a new round of discussions on the effects of omega-3 PUFA in prostate cancer [26, 187–195]. Even though many researchers hold the view that omega-3 PUFA intake is beneficial in this cancer type, the association between omega-3 PUFA and prostate cancer risk is not without controversy. On one hand, a case-control study with 3141 participants in Canada provided an inverse association between fish consumption and prostate cancer incidence and subsequently suggested that fish consumption may reduce the risk of developing prostate cancer [103]. Accordingly, a study focusing on aggressive prostate cancer also found that a high intake of omega-3 PUFA was strongly associated with a decreased risk of aggressive prostate cancer, and the odd ratio (OR) for prostate cancer comparing the highest with the lowest quartile of omega-3 intake was 0.37 (0.25–0.54) [106]. Moreover, Szymanski's meta-analysis showed an association between fish consumption and a significant 63% reduction in prostate cancer-specific mortality [116]. Chavarro et al. found that, compared to prostate cancer patients who consume fish less than once a week, patients who consume at least 5 times more fish per week had a 48% lower risk of prostate cancer death [108]. Torfadottir et al.'s study [110] on Icelanders found a negative correlation between risk of advanced prostate cancer and FO consumption in later life. Due to daily intake of omega-6 PUFA from the diet, the role of the omega-6/omega-3 PUFA ratio has also been considered. Williams et al. [104] found that the ratio of dietary omega-6/omega-3 PUFA was significantly associated with increased risk of high-grade, but not low-grade prostate cancer. Moreover, it has also been found [196, 197] that omega-3 PUFA or a low ratio of omega-6 to omega-3 PUFA could decrease the proliferation of prostate cancer cells in cell culture experiments. Based upon these association observations and considerable amounts of* in vivo* and* in vitro* experimental evidence [198, 199], several mechanistic hypotheses have been proposed to explain these phenomena. In particular, as already described in detail in Section 5.1, PUFA can be converted into many bioactive metabolites, which play various roles in modulating cell proliferation, apoptosis, and immune regulation. Many of the metabolites derived from omega-6 arachidonic acid (AA), including prostaglandin E2 and leukotriene B4, were shown to stimulate prostate cancer [200, 201].

On the other hand, several other studies did not provide evidence of a protective role of omega-3 against prostate cancer. Kristal et al.'s study [107] suggested that there was no association between omega-3 PUFA intake and prostate cancer risk. Brouwer's meta-analysis showed that the status of EPA or DHA did not associate with prostate cancer risk [117]. Park et al. [109] found no significant association between erythrocyte membrane omega-3 PUFA (EPA, DPA, or DHA) and total or advanced/high-grade prostate cancer risk. In Mannisto's study among male smokers [111], no association between serum EPA or DHA and prostate cancer risk was found. Harvei et al. [115] could not identify a clear association between prostate cancer risk and total serum phospholipid (PL) omega-3 PUFA either. A study based on the 2003–2010 National Health and Nutrition Examination Survey (NHANES) [114] found no association between omega-3 PUFA intake and prostate-specific antigen (PSA) level. Chua et al. [112] found that a high blood docosapentaenoic acid (DPA) level was negatively associated with total risk of prostate cancer. In contrast, a significant positive association between EPA or DHA and high-grade prostate cancer was shown [112]. Similarly, Sorongon-Legaspi et al.'s study [113] showed that DPA blood level was negatively linked with total prostate cancer risk and high EPA and DHA blood levels were associated with high-grade prostate cancer risk. Crowe et al. [105] described a positive association between EPA and risk of only high-grade prostate cancer, while DHA could not be linked to risk of any stages of prostate cancer.

As mentioned above, in their recently published case-control substudy from the SELECT study population Brasky et al. [25] reported a positive association between high plasma phospholipid omega-3 PUFA content at baseline and prostate cancer. However, we should underline that the SELECT trial was not addressing omega-3 PUFA supplementation for the prevention of prostate cancer. The authors used data from two case-control studies [25, 202], nested within intervention trials which were unrelated to eating fish or taking omega-3 supplements. In these studies, the dietary intake of fish or omega-3 had not been analyzed, neither advice was given to eat fish or take fish oil supplement [194]. Moreover, omega-3 PUFA were measured only once at baseline and only in plasma phospholipids [27] and not in red blood cell membranes. In fact, it has been suggested that evaluating the “omega-3 index,” that is, the incorporation of omega-3 PUFA in erythrocytes, may better reflect the omega-3 PUFA status of the subjects [77, 78].

The current state of evidence for the effects of omega-3 PUFA in human studies regarding prostate cancer and the contrasting outcomes have been summarized in Table 7, and additional possible reasons for the controversies in the field are reported in Section 6.

Overall, we can conclude that the literature on the role of omega-3 PUFA in prostate cancer is remarkably consistent among cell culture and animal studies, and controversies came mostly from association studies in human populations. However, the preventive effect of omega-3 PUFA on prostate cancer is still uncertain, and the controversy will probably remain until the mechanisms are clarified. However, while current human data are certainly not sufficient to recommend omega-3 PUFA to prevent prostate cancer, they are also far from being sufficient to support a prostate cancer promoting effect of these FA [188, 190, 191, 193, 203, 204].

## 6. Possible Reasons for Controversies

Several reasons may be responsible for the discrepancies registered among the outcomes of human studies. First of all, it should be considered that even though omega-3 PUFA can be regarded as medication and high-dose preparations are approved as prescription drugs in many parts of the world, omega-3 PUFA are essential part of our diet. Their intake can vary widely from population to population and even within the same person, depending on actual dietary habits or short-term dietary changes (eating seafood during vacations on the coast). Often, the epidemiological observational studies, particularly the early studies, refer to Food Frequency Questionnaire and Diet Records to establish fish consumption. However, this methodology is prone to measurement errors [205], and a low correlation coefficient (0.11–0.18) has been shown to exist between fish intake frequency and direct measurements of FA in patient samples [205, 206], even after adjusting for confounding factors, such as age [207, 208]. A number of other variables may contribute to this scarce correlation, such as the individual capacity to adsorb and make the PUFA bioavailable at the serum level, the fat content of the diet, or the omega-3/omega-6 PUFA dietary ratio. Moreover, different methods are used to calculate dietary fats or specific FA in the diets [209]. Often the questionnaires refer only to fish servings per week, without considering the serving size or the kind of fish consumed (lean fish or fatty fish) [207]. It is known that there exists an extreme variability in fish intake among populations, and if we consider the amount of fish ingested by the “low fish consumers” among the populations at high fish intake, it may correspond to or be even higher than the amount consumed by the “high fish consumers” in the populations at low fish intake. As a matter of fact, positive association between dietary intake of omega-3 PUFA and health effects has been registered mainly among population eating fish at high levels [15]. Some intrinsic weaknesses of questionnaire studies could thus be addressed by actually measuring omega-3 PUFA in the participants. Some more recent studies have directly evaluated the levels of omega-3 PUFA in serum, in different plasmatic lipid classes, or in erythrocytes [210–212]. Whereas it has been argued that measuring the omega-3 PUFA level only in one class of plasma lipids may be misleading [27], it has been suggested that erythrocyte EPA and DHA content (named “omega-3 index”) may better reflect the omega-3 PUFA status of the subjects [77, 78]. This is because the plasma-based measurements merely represent the short-term availability of omega-3 PUFA, being susceptible to artificial elevation following an acute omega-3 PUFA load [213]. Indeed, it has been recently reported that an acute single dose (3.4–4 g/d) of omega-3 PUFA, in the form of either prescription medication or dietary supplements, peaked plasma EPA + DHA levels as early as 5 h after administration, whereas the EPA plus DHA concentrations in erythrocyte membranes were only increased by approximately 10 orders of magnitude less than the concurrent plasma (3% versus 30%) from baseline at 24 h [214, 215]. Therefore, even if a single evaluation may be confounding, since serum FA are powerfully affected by feeding/fasting cycles and by the lipid content of the last meal consumed [27], one possibility of overcoming the discrepancies in omega-3 PUFA research is to call for actual correlation of questionnaire-based calculations of omega-3 PUFA intake with at least one actual measurement per participant in order to obtain some biochemical information regarding actual omega-3 PUFA levels.

Moreover, we ask if intervention studies administering defined amounts of omega-3 PUFA may solve this problem. At least these studies exactly define the amount of omega-3 PUFA administered. A rather big problem with these studies is the big range of doses of omega-3 PUFA administered on one hand and the variability of baseline diets on the other hand. In order to understand discrepancies and problems of omega-3 PUFA efficacy in intervention trials, we will have to regard the omega-3 PUFA as parts of diet and component of normal human body homeostasis. This is a concept obviously radically different from synthetic drug studies and something that has always to be taken into account when assessing intervention studies. In such studies, the animals/subjects have been treated with specific and controlled amounts of omega-3 PUFA. But most of these studies neither established baseline omega-3 PUFA body content in the participants nor monitored omega-3 PUFA during the study, making proper interpretation of the obtained results difficult. A recent study properly addressing these issues was the Welcome Trial in NASH patients, in which interpretation of the data focused on the correlation of measured DHA enrichment with liver fat content, as well as on pure ITT analysis of the treatment group. It demonstrated a statistically significant linear correlation between decreased liver fat content and increased DHA enrichment, but no significant effect in the ITT treatment group analysis [64]. Therefore, as in observational studies, intervention trials should perform biochemical analyses of omega-3 PUFA content to (1) define baseline levels in the different groups and (2) determine actual changes in the different groups due to the treatment as compared to the placebo group, where some of the participants might actually contaminate the results by starting to consume a high fish diet during the trial period.

Omega-3 PUFA dose is an issue as well. The omega-3 PUFA dose was very high in earlier animal studies, ranging from 2.0 to 8.0 (EPA + DHA g/kg body weight/day in mice) in most cancer studies [216]. But even the lower doses used for animals, after the interspecies conversion [217], appear rather high for humans (about 10–20 g/day in a person weighing 70 kg). However, it is remarkable that, at least in the few human interventional trials completed in the cancer field, the preventive effects were noticed with doses of only 2.0 g/day (corresponding to about 0.03 g/kg body weight in a person weighing 70 kg) [85, 91, 218, 219]. The positive anticancer effects were accompanied (if evaluated) by a comparable increase (ranging from 2-3- to 5-fold) in omega-3 PUFA incorporation in plasma or tissue lipids, in both animals and humans [216].

The source of the omega-3 PUFA ingested may also be important. Interventional studies often use highly purified omega-3 PUFA. They may help to obtain more clear results than the use of fish/FO, since fish also represents a good source of selenium, various vitamins, taurine, and other compounds that have been shown to possess potential protective effects [220–222]. Moreover, high levels of contaminant and dangerous agents may accumulate in fish tissues and fat. Some of these agents (such as organochlorine pesticides or Hg) are environment-derived and may be present in the tissues of both farmed and wild fish (especially predator fish). Moreover, other cytotoxic and carcinogenic products of omega-3 PUFA may be formed in fish after cooking them at extremely high temperature (by broiling/frying fish) or preserving them by smoking procedures [26]. The omega-3 PUFA oxidative products may be formed in omega-3 fortified food and in omega-3 supplements that do not contain adequate levels of antioxidants and are stored at room temperature for a long time [26]. Furthermore, the general composition of the diet has to be taken into account, since the effect of increased omega-3 PUFA uptake might be different in the context of a high-carbohydrate diet as compared to a high-fat diet. This might change absorption and processing of the omega-3 PUFA and thus affect actual concentrations in the body. In addition, the highly critical factor omega-6 PUFA and the actual omega-6/omega-3 PUFA dietary ratio may deeply alter the effects of omega-3 PUFA supplementation.

Considerable attention has been drawn recently to the possibility that interactions may exist between the subject genetic background and the effects of nutritional interventions [223, 224]. Different observational studies have already demonstrated that the inverse association between omega-3 PUFA intake or plasma/tissue enrichment and the risk of several diseases may be limited only to subpopulations possessing specific genetic features/signatures [99, 106, 225–230]. Genetic differences or gender-specific effects [97] might be due to differences in the expression of effector proteins for the omega-3 PUFA and their lipid mediator products, but also due to differences in enzymes of lipid metabolism that might lead to differences in the processing of the omega-3 PUFA in the body. Both issues, assessing the source of omega-3 PUFA and dietary composition as well as genetic background affecting handling of the omega-3 PUFA, also call for more measurements to better understand controversial results.

## 7. Conclusions

Our review of the omega-3 PUFA research literature prompts us to conclude that the animal and* in vitro* data have been remarkably consistent in showing health benefits, particularly through mechanisms dampening inflammation and proliferation in different tissues. These results have established potential protective effects of omega-3 PUFA in diseases ranging from cardiac arrhythmia and inflammatory conditions (atherosclerosis to airway inflammation, colitis, pancreatitis, steatohepatitis, arthritis, etc.) to tumorigenesis (particularly colon and liver tumors, but also breast and prostate cancer). On the other hand, the outcomes of human studies have been so far quite controversial. Therefore, in order to shed more light on this point and better understand if the divergences were ascribable to possible methodological weaknesses in human studies or, alternatively, to different responses of human cells/tissues to the incorporation of omega-3 as compared to those of laboratory animal species, it is suggested that all future studies in this field should perform blood FA measurements in trial participants. These evaluations should be preferably performed in blood cells, and, whenever possible, at baseline as well as at different time-points during the study. This procedure would also be extremely helpful to (1) monitor the effect of the huge omega-3 PUFA supplement industry in the western world on omega-3 PUFA content in humans, (2) understand the effect of other FA in the context of omega-3 PUFA interventions, and (3) recognize variations in individual responses to omega-3 PUFA supplementation.

## Figures and Tables

**Table 1:** Recommended dietary intakes for omega-3 fatty acids in adults from national and international organizations.

Region/country	Organization, year, reference	ALA (g/day)	DHA (g/day)	EPA + DHA (g/day)
*International *	FAO/WHO, 2010 [38]			0.25–2.0
	ISSFAL, 2004 [39]			>0.5
	Eurodiet, 2001 [40]	2.0		0.2
	WAPM, 2008 [41]		0.2–0.3	
	EFSA, 2010 [42]			0.25

*National *				
UK	SACN, 2004 [43]			0.45
Netherlands	Health Council, 2006 [44]			0.45
France	ANSES, 2001 [46]		0.25	0.5
			0.2	
Spain	SENC, 2011 [48]			0.5–1.0
Australia	NHMRC, 2006 [49]	M: 1.3 F: 0.8		M: 0.61 F: 0.43
USA	IoM, 2005 [50]	M: 1.6 F: 1.1		
	American Diabetes Association, 2007 [51]	2.0		0.2
	ADA USA & Canada, 2007 [52]	M: 1.6 F: 1.1		0.5
	AHA, 2009 [53]			0.5–1.0

M: male; F: female.

**Table 2:** Recommended dietary intakes for omega-3 fatty acids in children from national and international organizations.

Region/country	Organization, year	Age range	ALA (mg/day)	DHA (mg/day)	EPA + DHA (mg/day)
*International *	FAO/WHO, 2010 [38]	6–24 mo		10–12/kg	
		2–4 y			100–150
		4–6 y			150–200
		6–10 y			200–250
	EFSA, 2010 [42]	7–24 mo		100	
		2–18 y			250

*National *					
Belgium	CSS, 2009 [47]	0–12 mo	500		
Netherlands	Health Council, 2001 [45]	0–5 mo	80/kg	20/kg	
		6 mo–18 y			150–200
France	ANSES, 2001 [46]	6 mo–3 y		70	
		3–9 y		125	250
		10–18 y		250	500
Australia	NHMRC, 2006 [49]	1–3 y	500		40
		4–8 y	800		55
		9–13 y	M: 1000 F: 800		70
		14–18 y	M: 1200 F: 800		M: 125 F: 85
USA	IoM, 2005 [50]	1–3 y	700		
		4–8 y	900		
		9–13 y	M: 1200 F: 1000		
		14–18 y	M: 1600 F: 1100		

M: male; F: female.

**Table 3:** Current state of evidence for the effects of omega-3 PUFA in published human intervention studies regarding cardiovascular disease.

Type of study	Beneficial effect	Beneficial effect limited to subpopulation	Detrimental effect	No effect
Clinical trials	Lower risk of cardiovascular events and death with open-label omega-3 PUFA treatment (850 mg/d) [18]	High-dose omega-3 PUFA intervention in ICD patients at high risk of arrhythmia—significant protection in patients treated per protocol for 11 months [54]		Administration of 900 mg/d omega-3 PUFA in dysglycemic patients at increased cardiovascular risk had no protective effect [23]
	Lower risk of death in patients with heart failure on 1 g/d omega-3 PUFA [55]			No decrease in major cardiovascular events in patients on 500 mg/d omega-3 PUFA [56]
	Cardioprotective effect of open-label EPA supplementation in addition to a statin in hypercholesterolemic patients [57]			Administration of 1 g/d omega-3 PUFA in patients after a myocardial infarction showed no benefit [58]
				No reduction in cardiovascular events in patients after myocardial infarction with either 400 mg/d EPA + DHA or 2 g/d ALA, or both [21]
				No VT/VF protection in ICD patients on 1300 mg/d omega-3 PUFA [59]
				No arrhythmia protection in ICD patients on 2 g/d fish oil [60]

Meta-analyses				Current data do not support the concept of increasing omega-3 PUFA or omega-6 PUFA or decreasing saturated fatty acid intake, to reduce cardiovascular risk [61]
				No benefit of omega-3 PUFA supplementation in 14 randomized double-blind placebo-controlled studies [22]
				No benefit of omega-3 PUFA supplementation in randomized clinical trials assessing cardiovascular endpoints [62]

ICD: implanted cardioverter/defibrillator; VF: ventricular fibrillation; VT: ventricular tachycardia.

**Table 4:** Current state of evidence for the effects of omega-3 PUFA in human studies regarding gastrointestinal inflammatory conditions.

Type of study	Beneficial effect	Beneficial effect limited to subpopulation	Detrimental effect	No effect
Clinical trials	Reduced rate of relapse with 2.7 g/d omega-3 PUFA in patients with Crohn's disease in a double-blind placebo-controlled study in 78 patients [63]	Decreased liver fat content with increased DHA enrichment in NASH patients [64]		Randomized, double-blind, placebo-controlled treatment with approx. 4 g/d omega-3 PUFA did not prevent relapses in patients with Crohn's disease [65]
	Improvements in histologic findings and weight gain in 18 ulcerative colitis patients on 5 g/d EPA + DHA in a randomized double-blind placebo-controlled crossover trial [66]			
	Shortened hospital stay in an open-label randomized prospective study administering 3.3 g/d enteral fish oil in patients with acute pancreatitis [67]			

Meta-analyses				Current data are not sufficient to support the concept of omega-3 PUFA supplementation for the treatment of inflammatory bowel disease [68]

NASH: nonalcoholic steatohepatitis.

**(a):** 

Type of study	Enrolled subjects	Number of subjects	Omega-3 PUFA daily treatments	Control group daily treatment	Length of treatments	Antineoplastic effect observed in omega-3 PUFA-treated subjects	Reference
Randomized double-blind, placebo-controlled trial	High risk for CRC (sporadic adenomatous polyps)	10 (control group) 10 (omega-3 PUFA group)	OS: 4.1 g EPA-EE + 3.6 g DHA-EE	OS: 7 g olive oil	2 wk.–3 mo.	Inhibition of abnormal rectal mucosa cell proliferation	[83]

Double-blind crossover trial	Healthy volunteers	12 (control group) 12 (omega-3 PUFA group)^*∗*^	OS: 11 g FO (providing 4.4 g omega-3 PUFA); omega-3/omega-6 ratio in the basal diet: 0.40	11 g corn oil	2–4 wk.	Inhibition of rectal mucosa cell proliferation; inhibition of PGE2 release by rectal biopsies	[84]

Randomized double-blind, placebo-controlled trial	High risk for CRC (sporadic adenomatous polyps)	10 (control group) 10 (omega-3 PUFA group)	OS: 2.5 g EPA-EE + DHA-EE	2.5 g olive oil	1–6 mo.	Inhibition of abnormal rectal mucosa cell proliferation (in subjects with abnormal baseline proliferation pattern)	[85]

Double-blind crossover trial	Healthy volunteers	12 (control group) 12 (omega-3 PUFA group)^*∗*^	OS: 11 g FO (providing 4.4 g omega-3 PUFA); omega-3/omega-6 ratio in the basal high-fat diet: 0.25	11 g corn oil	2–4 wk.	No effect on inhibition of rectal mucosa cell proliferation	[86]

Randomized double-blind, placebo-controlled trial	Patients with resected CRC or severely dysplastic polyps	10 (control group) 17 (omega-3 PUFA group)	OS: 9 g omega-3 PUFA-EE concentrate (providing about 4.0 g EPA-EE and 2.2 g DHA-EE)	9 g omega-6 PUFA-EE concentrate (providing about 4.5 g LA-EE)	6 mo.	Inverse association between colon omega-3/omega-6 PUFA and cell proliferation (in subjects with abnormal baseline proliferation pattern); suppression of polyp development	[87]

CRC: colorectal cancer; EE: ethyl ester; FAP: familial adenomatous polyposis; FFA: free fatty acid; FO: fish oil; LA: linoleic acid; OS: oral supplementation; TG: triglycerides.

^*∗*^The same subjects were treated with FO or CO in different periods.

**(b):** 

Type of study	Enrolled subjects	Number of subjects	Omega-3 PUFA daily OS treatments	Control group daily OS treatment	Length of treatments	Antineoplastic effect observed in omega-3 PUFA-treated subjects	Reference
Randomized double-blind, placebo-controlled trial	Patients undergoing surgery for CRC	24 (control group) 25 (omega-3 PUFA group)	2 g FO (providing 1.4 EPA + 1.0 DHA g n-3 PUFA)	2 g safflower oil	12 days prior to surgery	No effect on frequency and spatial distribution of crypt cell mitosis	[88]

Single-blind (investigators) trial	Patients polypectomized for CR adenomas/tumors	20 (control group) 21 (omega-3 PUFA group)	FO (providing 0.1 g EPA + 0.4 g DHA) and DA^*∗*^	No treatment; DA: reduction of fat consumption	2 years	Increased apoptosis in normal sigmoid colon mucosa	[89]

Randomized single-blind (investigators) trial	Patients with one or more CR adenomas	15 (control group) 15 (omega-3 PUFA group)	2 g enteric coated EPA-FFA formulation	No treatment; DA: no fish intake	3 mo.	Reduced cell proliferation and increased apoptosis in normal colon mucosa crypts	[90]

Phase III randomised, double-blind, placebo-controlled trial	FAP patients	27 (control group) 28 (omega-3 PUFA group)	2 g enteric coated EPA-FFA formulation	2 g capric and caprylic acid medium-chain TG	6 mo.	Reduced polyp number and diameter	[91]

Phase II double-blind, randomised, placebo-controlled trial	Patients carrying CRC liver metastases	35 (control group) 36 (omega-3 PUFA group)	2 g enteric coated EPA-FFA formulation	2 g capric and caprylic acid medium-chain TG	12–65 days (median 30 days) prior to surgery	Reduced vascularity of CRC liver metastases	[92]

CRC: colorectal cancer; DA: dietary advice; FAP: familial adenomatous polyposis; FFA: free fatty acid; FO: fish oil; OS: oral supplementation; TG: triglycerides.

^*∗*^DA: reduction of fat intake and increase in omega-3 PUFA/omega-6 PUFA dietary ratio.

**Table 6:** Current state of evidence for the effects of omega-3 PUFA in published human studies regarding colorectal cancer.

Type of study	Beneficial effect	Beneficial effect limited to subpopulation	Detrimental effect limited to subpopulations	No effect
Clinical trials	Antiproliferative effect [83–85, 87, 90]			No antiproliferative effect [86, 88, 93, 94]
	Proapoptotic effect [89, 90]			No proapoptotic effect [93, 94]
	Reduced polyp number and size in FAP [91]			No anti-inflammatory effect in colony biopsies [93, 94]
	Reduced angiogenesis [92]			
	Reduced CRP levels in serum [93–95]			

Observational studies		Inverse association between increased dietary intake and risk of CR adenomas (only in women) [96]	Positive associations between FO use and CRC in high risk groups [97]	
		Inverse association between FO use and cancer risk (in men, not in women; in colon, not in rectum) [97]	Positive association between increased intake and distal CC [98]	
		Inverse associations between FO use and CRC in low-moderate genetic risk groups, and positive associations among high risk groups [97]		
		Inverse association between increased intake and RC (but not CC) [98]		
		Inverse association between increased dietary intake and risk of CRC (only in specific genetic variants) [99]		

Meta-analyses	Significant inverse association between fish consumption and RC [100]			Limited evidence of a role in CRC prevention [101, 102]

CC: colon cancer; CRC: colorectal cancer; CRP: C-Reactive Protein; FAP: familial adenomatous polyposis; FO: fish oil; RC: rectal cancer.

**Table 7:** Current state of evidence for the effects of omega-3 PUFA in published human studies regarding prostate cancer.

Type of study	Beneficial effect	Beneficial effect limited to subpopulations	Detrimental effect limited to subpopulations	No or detrimental effects
Observational studies	Inverse association between fish consumption and cancer incidence [103]	Dietary omega-3/omega-6 PUFA ratio inversely associated with risk of high-grade cancer [104]	Positive association between EPA and risk of only high-grade cancer [105]	Dietary omega-3/omega-6 PUFA ratio not associated with risk of low-grade cancer [104]
	Inverse association between high intake and risk of aggressive cancer [106]		Positive association between high serum PL LC-omega-3 PUFA and cancer risk [25]	No association between fish or FO intake and cancer risk [107]
	Inverse association between higher fish intake and risk of cancer death [108]			No association between erythrocyte membrane EPA, DPA, or DHA and total or advanced/high-grade cancer risk [109]
	Inverse association between FO intake and risk of advanced cancer later life [110]			No association between serum EPA or DHA and cancer risk in male smokers [111]
	Inverse association between DPA level and total risk of cancer [112, 113]			No association between omega-3 PUFA intake and PSA level [114]
				Positive association between EPA or DHA and high-grade cancer [112, 113]
				No association between DHA level and risk of cancer at any stages [105]
				No association between total serum PL omega-3 PUFA and cancer risk [115]

Meta-analyses	Inverse association between fish intake and prostate cancer-specific mortality [116]			No association between FO intake or EPA/DHA blood level and cancer risk [117]

FO: fish oil; PL: phospholipids; PSA: prostate-specific antigen.
